# Complete chloroplast genome analysis of *Polygala chinensis* L. (= *P. glomerata* Lour.) (Fabales, Polygalaceae)

**DOI:** 10.1080/23802359.2026.2617765

**Published:** 2026-01-19

**Authors:** Chao Chen, Pin Lan, Yuan-Hua Fang, Lin-Jie Lai, Jie Chen, Wan-Juan Liu

**Affiliations:** a Emergency Medical Center, Fifth Affiliated Hospital of Wenzhou Medical University, Lishui, China; b Industrial College of Traditional Chinese Medicine and Health, Lishui University, Lishui, China

**Keywords:** Medicinal plant, genomic resource, phylogenetic relationship

## Abstract

*Polygala chinensis* L. is a medicinal herb with anti-inflammatory activity and traditional use against snake envenomation. We assembled and annotated its complete chloroplast genome using Illumina data. The chloroplast genome is 163,097 bp and exhibits a typical quadripartite structure, comprising a large single-copy region (83,285 bp), two inverted repeats (34,925 bp each), and a small single-copy region (9,962 bp). It contains 113 unique genes, including 78 protein-coding genes, 30 tRNA genes, and 5 rRNA genes. Phylogenetic analysis placed *P. chinensis* as sister to *Polygala subopposita*. This work provides the first species-specific chloroplast genome reference for *P. chinensis*, enabling precise molecular identification and evolutionary studies.

## Introduction

*Polygala chinensis* L. (1753; syn. *P. glomerata* Lour., 1790), a herbaceous milkwort in the family Polygalaceae, is widely distributed across tropical and subtropical Asia, extending from southern China and India through mainland Southeast Asia to northern Australia. Occupying open grasslands and roadside margins, its dense low canopy helps stabilize disturbed soils during early-successional stages, thereby reducing erosion on exposed sites.

Ethnopharmacological records show that the whole plant has long been employed in traditional medicine as an anti-inflammatory tonic and, notably, as a topical or decocted remedy for snake-bite envenomation. Modern pharmacology confirms significant anti-inflammatory activity of ethanolic extracts in carrageenan-induced paw-oedema assays (Alagammal et al. 2012). GC-MS profiling has further revealed a rich spectrum of lignans, flavonoids and fatty acids that are thought to underlie these therapeutic effects and have contributed to detailed morphological and chemical characterization of the species (Alagammal et al. 2011).

Although such morphological and phytochemical traits are well documented, genomic resources for *P. chinensis* remain scarce. To date, complete chloroplast genomes have been reported for only a handful of congeners, such as *P. subopposita* (Wang 2023) and *P. tatarinowii* (Wang 2025)—and fewer than twenty chloroplast genomes are available for the entire family. The absence of a species-specific chloroplast genome has hindered finer-scale phylogenetic inference, barcoding, and exploration of the genetic basis for the medicinal properties of *P. chinensis*.

In the present study we sequenced, assembled and annotated the complete chloroplast genome of *P. chinensis*. This new genomic resource provides a critical benchmark for comparative analyses within Polygalaceae, facilitates accurate molecular identification of medicinal material, and lays the groundwork for future studies on the evolution and pharmacological exploitation of this widely used snake‑bite remedy.

## Materials and methods

**Figure 1.**
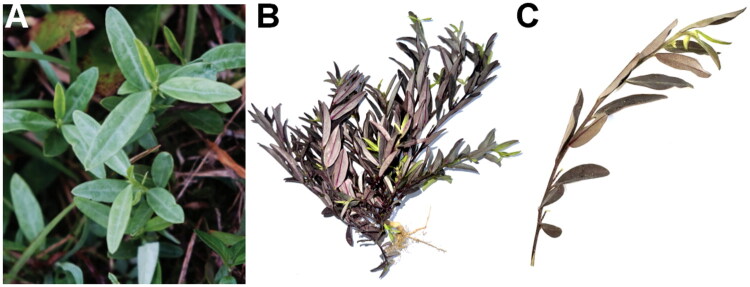
*Polygala chinensis*. (A) Living plant in its natural habitat (*in situ*). (B) Freshly collected whole plant before pressing. (C) Close-up of a vegetative shoot highlighting diagnostic features. Photographs taken by the author, Wan-Juan liu.

*P. chinensis* was collected on a gentle hillside near Maoming City, Guangdong, China, in a semi-open grassland with scattered shrubs at the forest margin (22.18°N, 111.17°E). This location matches the typical habitat of this species in grasslands, shrub forests, and hill slopes across subtropical Asia. The species was identified as *P. chinensis* by Wan-Juan Liu based on diagnostic morphological characters. A single voucher specimen (2025-02-052) is archived at the Fifth Affiliated Hospital of Wenzhou Medical University (Contact: Wan-Juan Liu, lwj_lch@126.com; Figure 1).

Genomic DNA was extracted from leaves using a Rapid Plant Genomic DNA Isolation Kit (Sangon, Shanghai, China). Subsequent sequencing on the Illumina HiSeq 2500 platform produced 150-bp paired-end reads. These reads were screened and assembled into a genome using GetOrganelle (Jin et al. 2020). Coverage depth of the assembled genome was calculated with SAMtools v1.16.1 (Li et al. 2009), and the depth/coverage profile was visualized using the ggplot2 package (Ito and Murphy 2013) in R (Figure S1). Annotation of the chloroplast genome was carried out using the online tools GeSeq (Tillich et al. 2017) and CPGAVAS2 (Shi et al. 2019), which were used to determine the start positions of genes, as well as the boundaries of the inverted repeats (IR) and other gene loci. Finally, CPGView (Liu et al. 2023) was employed to refine the annotation, visualize the chloroplast genome architecture, and characterize gene structures encompassing cis-splicing and trans-splicing.

Phylogenetic relationships among 15 Fabales species were inferred from chloroplast genome sequences. *Gleditsia microphylla* (Fabaceae) served as the outgroup taxon, selected based on its phylogenetic proximity to (but exclusion from) the *Polygala* ingroup—a common reference point in Fabales chloroplast studies. Its appropriateness as an external reference is confirmed by Xiao et al. (2024), who completed its chloroplast genome sequencing and utilized it in broader *Gleditsia* genus analyses. Sequence alignment was performed using MUSCLE version 3.8.31 (default options; Edgar 2004). Tree building under the maximum likelihood criterion utilized RAxML software (Stamatakis 2006), incorporating the GTR + G + I evolutionary model and 1000 bootstrap iterations. The phylogenetic tree was rendered graphically using ggtree (Xu et al. 2022) within the R environment.

## Results

**Figure 2.**
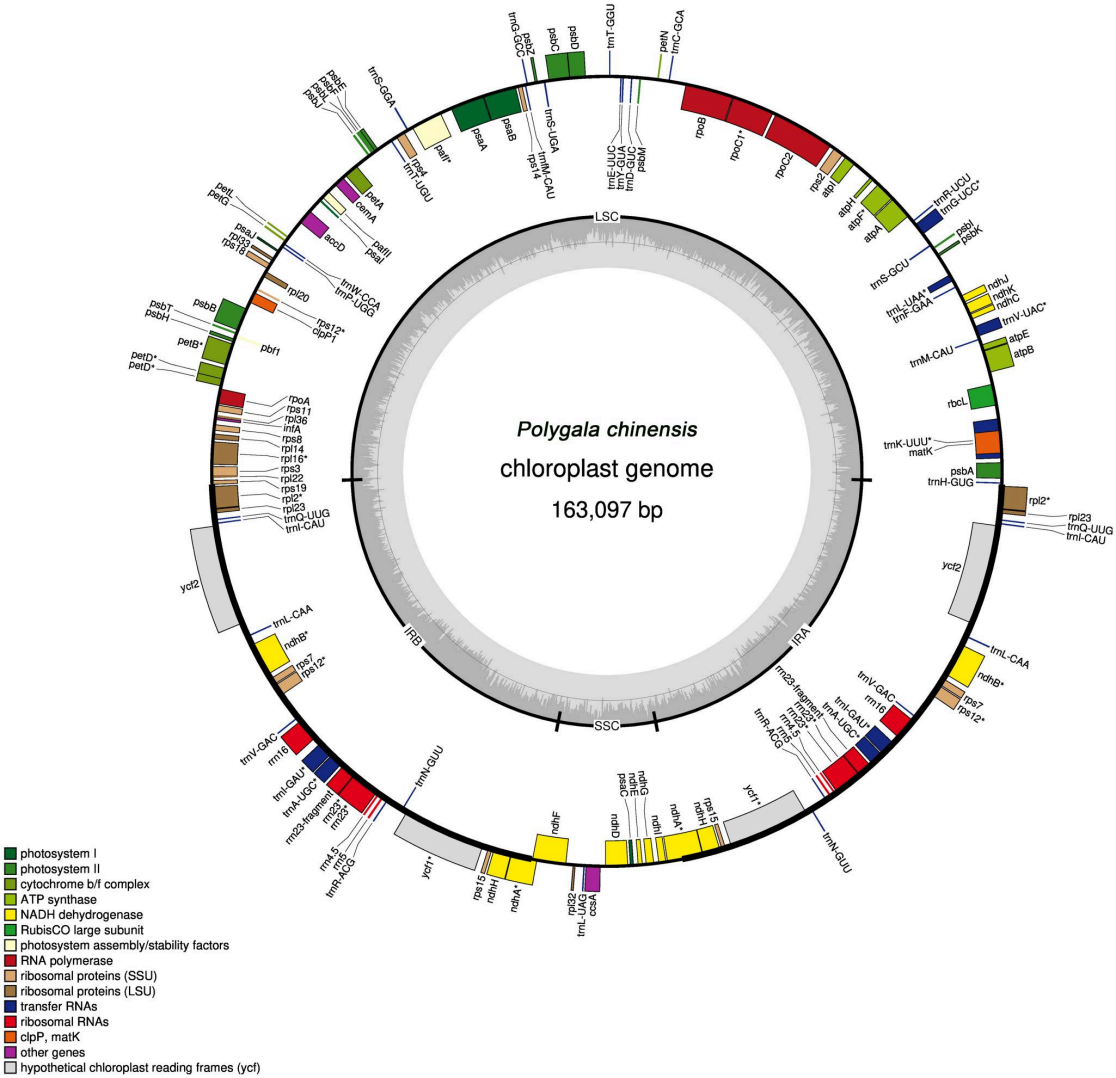
*Polygala chinensis* chloroplast genome map. The inner circle shows the GC (dark grey) and AT (light grey) contents. Different colors on the outer circle indicate different functional gene classifications. Asterisk (*) indicates intron-containing genes.

The complete chloroplast genome of *P. chinensis* measures 163,097 bp, exhibiting an overall GC content of 36.68%. Its quadripartite structure consists of an 83,285 bp large single-copy (LSC) region, two 34,925 bp inverted repeats (IRA and IRB), and a 9,962 bp small single-copy (SSC) region (Figure 2). Annotation revealed 113 unique genes: 78 protein-coding genes (PCGs), 30 transfer RNA genes, and 5 ribosomal RNA genes. Multiple sequence alignments confirmed ten cis-spliced genes (*pafI*, *rpl2*, *rpl16*, *petD*, *petB*, *atpF*, *rpoC1*, *ycf1*, *ndhB*, and *ndhA*; Figure S2). Additionally, the *rps12* gene displays a characteristic split structure, a feature commonly observed in plant chloroplast genomes (Figure 2; Figure S3).

**Figure 3.**
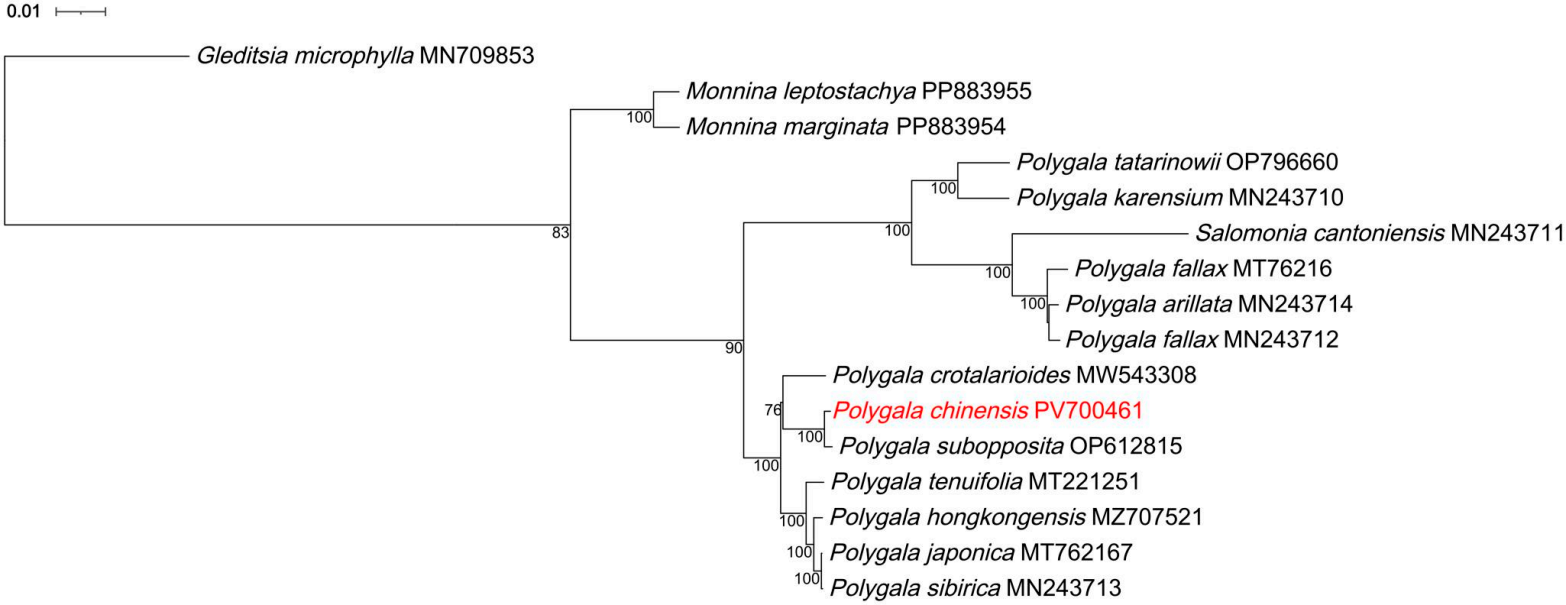
Phylogenetic tree based on chloroplast genome sequences of *polygala chinensis* and 14 related fabales species. *Gleditsia microphylla* was used as the outgroup to root the tree. The chloroplast genome generated in this study is highlighted in red. Bootstrap values are indicated at the nodes. The horizontal scale denotes genetic distance, with one unit equivalent to 0.01. GenBank accession numbers for the chloroplast genome sequences of all species are shown in Table S1.

The maximum likelihood phylogeny (Figure 3) clearly separated the outgroup *G. microphylla* from the ingroup. Within the ingroup, *Monnina leptostachya* and *M. marginata* formed a strongly supported sister pair (bootstrap = 100). The *Polygala* clade (including *Salomonia*) was divided into two major lineages (bootstrap = 90). Our sample *P. chinensis* was recovered as sister to *P. subopposita* with maximal support (bootstrap = 100), and this pair clustered with *P. crotalarioides*.

## Discussion and conclusion

The 163,097 bp chloroplast genome of *P. chinensis* is architecturally conservative within the family Polygalaceae, yet it exhibits subtle, lineage-specific traits. Its overall GC content (36.68%) and gene complement (78 PCGs, 5 rRNAs, 30 tRNAs) closely mirror those of *P. subopposita* (164,784 bp; 77 PCGs) (Wang 2023) and *P. tatarinowii* (168,779 bp; 78 PCGs) (Wang 2025). The chloroplast genome of *P. chinensis* has an overall GC content of 36.68%, which is essentially identical to that of *P. tatarinowii* (36.8%) (Wang 2025) and *P. subopposita* (36.6%) (Wang 2023).

Phylogenetically, maximum-likelihood analysis firmly nests *P. chinensis* within the herbaceous milkwort clade and resolves it as sister to *P. subopposita* with maximal support (bootstrap = 100), and this pair further groups with *P. crotalarioides*. This topology is congruent with plastid and nuclear ITS studies that indicate rapid radiation of East-Asian *Polygala* lineages (Persson 2001). However, the placements of *S. cantoniensis* (a different genus within Polygalaceae) and the two *P. fallax* accessions appear unstable/dubious across analyses (e.g. distance-based trees versus maximum likelihood inference), and we therefore refrain from drawing genus-level conclusions or over-interpreting these sister relationships based on the current taxon sampling. A broader sampling of Polygalaceae plastomes and complementary nuclear data will be necessary to reassess these relationships.

Beyond systematics, a high-quality chloroplast genome delivers tangible applied benefits. *Polygala* preparations remain frontline ethnomedicines for inflammation and snake-bite envenomation, activities linked to lignan-and flavonoid-rich extracts (Alagammal et al. 2012). The complete chloroplast sequence now provides a reference for designing robust barcodes that enable accurate molecular authentication of *P. chinensis* and facilitate detection of potential substitution/mislabelling in medicinal materials, enhancing quality control in phytopharmaceutical supply chains.

In conclusion, the *P. chinensis* chloroplast genome bridges a major taxonomic gap in Polygalaceae genomic resources and furnishes a versatile platform for evolutionary, ecological and medicinal research. Our phylogenetic inference supports a close relationship between *P. chinensis* and *P. subopposita*, but the positions of *S. cantoniensis* and the two *P. fallax* accessions should be treated with caution until denser sampling and nuclear evidence are incorporated. Future work integrating population-level plastome variation with nuclear genomes and transcriptomes will be crucial for elucidating the genetic basis of this species’ adaptive traits and therapeutic potential.

## Supplementary Material

Figure_S1[1].tif

Figure_S3[1].tif

Figure_S2[1].tif

## Data Availability

The genome sequence data supporting this study are openly available in GenBank of NCBI at https://www.ncbi.nlm.nih.gov under the accession number PV700461. The associated BioProject, SRA, and Biosample numbers are PRJNA1269260, SRR33742878, and SAMN48783620, respectively.
