# Supplementary figures and images for: Complete chloroplast genome analysis of Polygala chinensis L. (= P. glomerata Lour.) (Fabales, Polygalaceae)
Source: Mitochondrial DNA B Resour. 2026 Jan 19;11(2):268–71. doi: 10.1080/23802359.2026.2617765 (PMC12818286; doi:10.1080/23802359.2026.2617765)

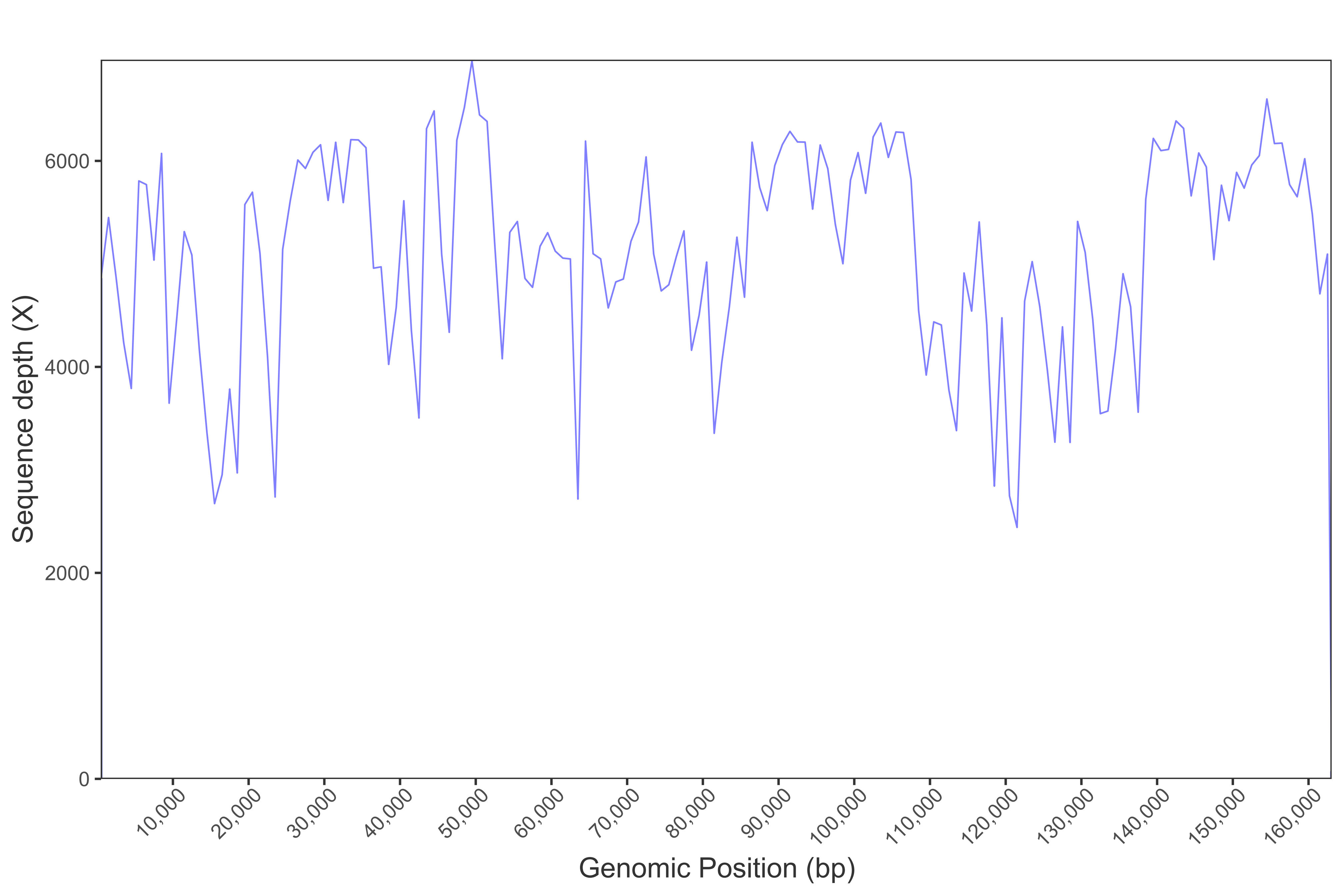

Supplement: Figure_S1[1].tif [file TMDN_A_2617765_SM9587.tif]

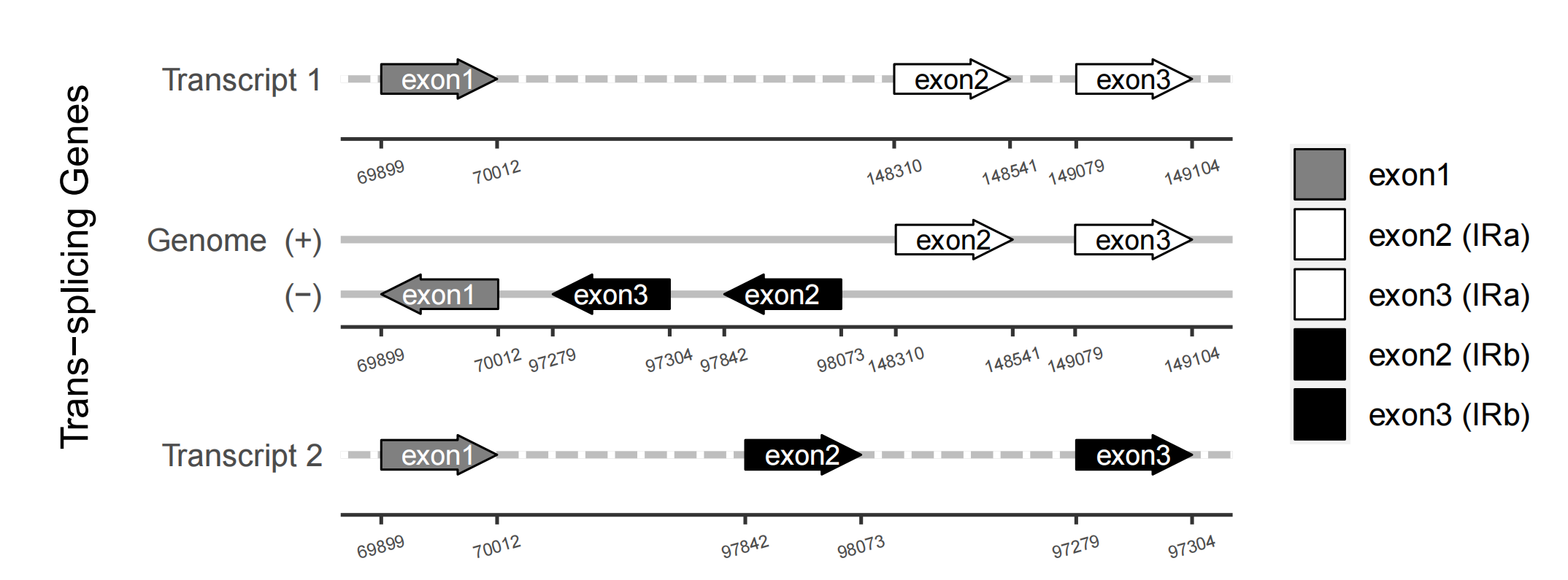

Supplement: Figure_S3[1].tif [file TMDN_A_2617765_SM9586.tif]

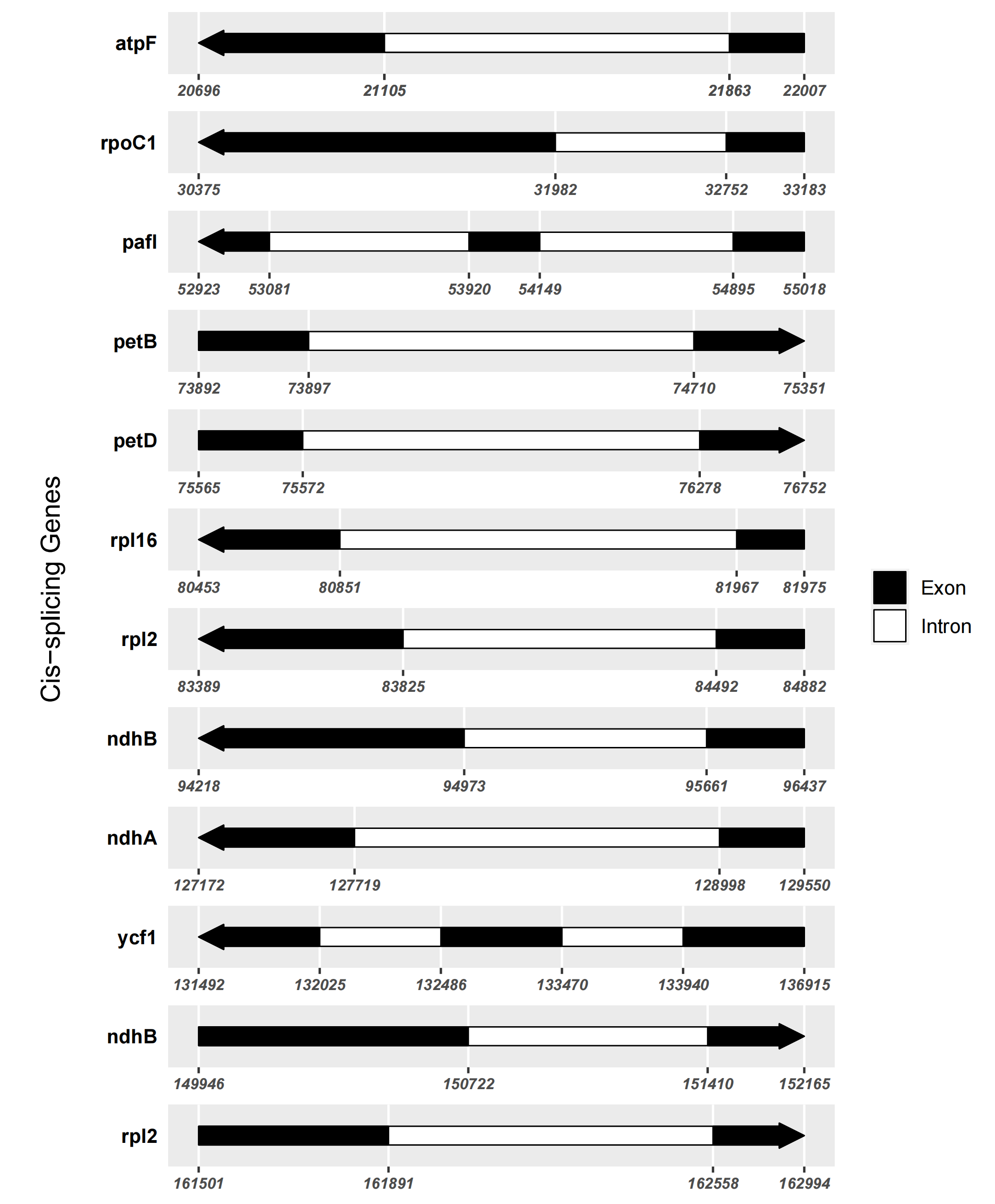

Supplement: Figure_S2[1].tif [file TMDN_A_2617765_SM9585.tif]
